# Targeting CLDN18.2 in cancers of the gastrointestinal tract: New drugs and new indications

**DOI:** 10.3389/fonc.2023.1132319

**Published:** 2023-03-10

**Authors:** Jinxia Chen, Zhiyuan Xu, Can Hu, Shengjie Zhang, Mengli Zi, Li Yuan, Xiangdong Cheng

**Affiliations:** ^1^ Department of Gastric Surgery, The Cancer Hospital of the University of Chinese Academy of Sciences (Zhejiang Cancer Hospital), Institutes of Basic Medicine and Cancer (IBMC), Chinese Academy of Sciences, Hangzhou, China; ^2^ Zhejiang Provincial Research Center for Upper Gastrointestinal Tract Cancer, Zhejiang Cancer Hospital, Hangzhou, China; ^3^ Zhejiang Key Lab of Prevention, Diagnosis and Therapy of Upper Gastrointestinal Cancer, Zhejiang Cancer Hospital, Hangzhou, China

**Keywords:** Claudin-18.2, gastrointestinal (GI) tract, monoclonal antibody, antibody–drug conjugate (ADC), chimeric antigen receptor T (CAR-T)

## Abstract

Cancers of the gastrointestinal (GI) tract greatly contribute to the global cancer burden and cancer-related death. Claudin-18.2(CLDN18.2), a transmembrane protein, is a major component of tight junctions and plays an important role in the maintenance of barrier function. Its characteristic widespread expression in tumour tissues and its exposed extracellular loops make it an ideal target for researchers to develop targeted strategies and immunotherapies for cancers of the GI tract. In the present review, we focus on the expression pattern of CLDN18.2 and its clinical significance in GI cancer. We also discuss the tumour-promoting and/or tumour-inhibiting functions of CLDN18.2, the mechanisms regulating its expression, and the current progress regarding the development of drugs targeting CLDN18.2 in clinical research.

## 1 Introduction

Cancers of the gastrointestinal (GI) tract accounted for an estimated 4.8 million new cancer cases and 3.4 million cancer-related deaths worldwide in 2018; these numbers correspond to over one-quarter (26%) of global cancer cases and over one-third (35%) of all cancer-related deaths (1). Thus, cancers of the GI tract remain important contributors to the global cancer burden. Improving the efficacy of tumour treatment is the key to improving the survival and prognosis of patients with GI tumours.

In tumour treatment, surgery and chemotherapy are the common treatment approaches (2). For patients with early cancer, surgical resection remains the primary treatment option; for patients with advanced metastasis or patients who cannot undergo surgical resection, chemotherapy is the most indispensable treatment (3). However, chemotherapy resistance and tumour progression and recurrence in cancer patients have become the most common causes of treatment failure (4). In the past decade, remarkable advances made by studies of tumour biology, tumour immunotherapy and targeted therapy have revolutionised the treatment of a fair number of cancers (5). Small molecule targeted drugs have shown certain advantages in tumour treatment.

Recently, increasing attention has been given to a potential target, Claudin18.2 (CLDN18.2). Its restricted expression in normal tissues and specific expression in tumour tissues makes it an ideal target for researchers to develop targeted therapy and immunotherapies for solid tumours (6). CLDN18.2 was initially identified as a gastric tumour-specific target, and many clinical trials of monoclonal antibodies targeting CLDN18.2 have been conducted for the treatment of patients with gastric and gastroesophageal cancer. However, several other novel targeting methods, including chimeric antigen receptor T (CAR-T) therapy, bispecific antibodies and antibody–drug conjugates (ADCs), have rapidly emerged in recent years, and a variety of corresponding clinical trials that have broadened their treatment adaptation to populations, especially those with GI tract cancer, have been conducted. This review will discuss the expression pattern of CLDN18.2, its roles in tumours and its relevant molecular mechanisms, as well as the current progress in the development of drugs targeting CLDN18.2 in clinical research.

## 2 Basic information of CLDN18

**Figure 1:**
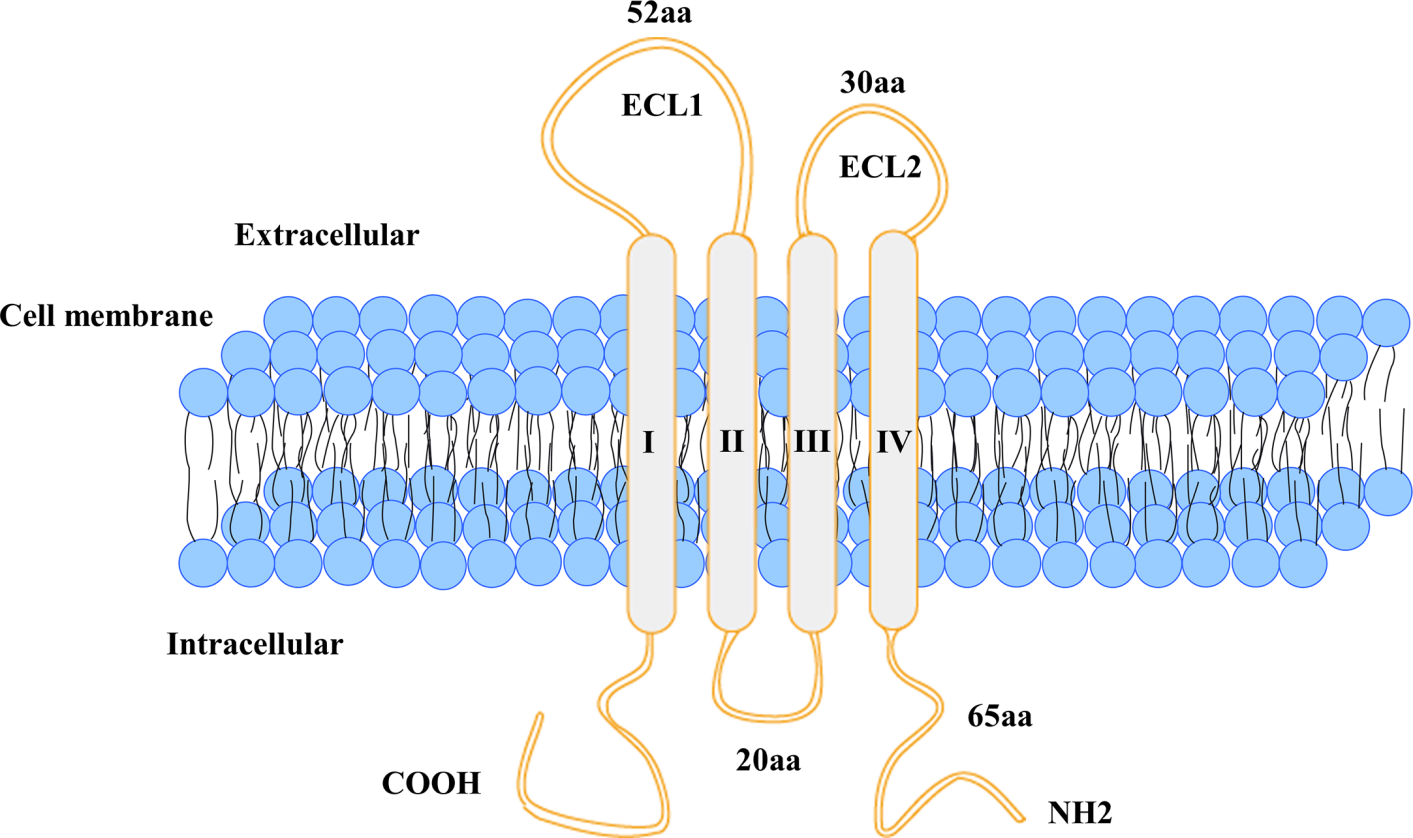
Schematic structure of CLDN18 protein.

Claudins, a family of at least 27 transmembrane proteins, are important components and functional structures of tight cell junctions. Claudin-18 is a major component of tight junctions located on the cell membrane surface; it plays an important role in the maintenance of cell polarity and barrier function and promotes acid resistance (7–10). For example, in the stomach, claudin-18 normally forms a paracellular barrier against H(+), promotes acid resistance, and causes paracellular H(+) leakage, persistent upregulation of proinflammatory cytokines and atrophic gastritis in mice (11). The human CLDN18 gene locus on chromosome 3q22 has a molecular weight of approximately 35 kb and contains 6 exons and 5 introns. The first exon of CLDN18 can be alternatively spliced, forming two different splice mutants (CLDN18.1 and CLDN18.2) that have highly homologous amino acid sequences (12). The transmembrane protein CLDN18 consists of two extracellular loops (ECLs), four transmembrane domains and a cytoplasmic domain. Both the C-terminus and the N-terminus of CLDN 18 are located in the cytoplasm (**Figure 1**) (6). Two CLDN18 protein isoforms are expressed in a tissue-specific manner—CLDN18.1 and CLDN18.2 are specifically expressed in normal stomach and lung tissues, respectively (13). CLDN18 is also expressed in cancer tissues and has altered functions that are linked to tumour formation, proliferation, invasion and migration (6, 14–18).

CLDN18.1 is highly expressed in lung alveolar epithelium and is involved in regulating solute and ion permeability, as well as cytoskeletal rearrangement (9). It has been found that CLDN18.1 knockout increased solute permeability and alveolar fluid clearance, and enhanced epithelial sodium channel and Na-K-ATPase activity (9). However, CLDN18.1 expression was significantly decreased in lung cancer and may play a role in inhibiting tumour progression (19, 20). Luo et al. found that when restored in lung adenocarcinoma (LUAD) cells that have lost expression, CLDN18.1 markedly attenuates tumour growth *in vivo* as well as cell proliferation, migration, invasion and colony formation *in vitro* (20). Zhou et al. also elucidated that knockout of Cldn18.1 results in increased lung size, progenitor cell proliferation, and tumourgenesis in mice (17).

CLDN18.2 is also involved in regulating ion permeability including H(+) and other cations (11). CLDN18.2 deficiency leads to neutrophils recruiment and inflammation development (11). As the focus of our review, the expression pattern and role of CLDN18.2 in tumours will be elaborated in detail later in the article.

ARHGAP26 is a GTPase-activating protein (GAP) that promotes conversion of RHO GTPases to the GDP state and has been indicated in enhancing cellular motility (21). CLDN18-ARHGAP26 fusion gene is one of the most frequent somatic genomic rearrangements in gastric cancer, especially in the genomically stable subtype (22, 23). Cell survival and migration studies performed by Ushiku et al. showed that CLDN18-ARHGAP fusions complemented cancer cell survival and induced cell migration under RHOA knockdown condition. RHOA mutations exist specifically in diffuse type gastric cancers (DGC) and are considered one of the DGC driver genes. Thus, CLDN18-ARHGAP fusion is possible a factor for RHOA mutation to maintain cancer cell survival and promoted cell migration (24).

## 3 The expression pattern and clinical significance of CLDN18.2 in cancers of the GI tract

For a long time, CLDN18.2 has been widely studied in patients with various solid tumours, especially in patients with cancers of the GI tract. Data for the expression pattern and clinical significance of CLDN18.2 in cancers of the GI tract are summarised in **Table 1**.

**Table 1:** The expression and clinical significance of CLDN18 in GI tumours.

Target	Country	Tumour type	Cases	Expression rate	High expression rate	Expression changes	Clinical significance	References
CLDN18.2	South Korea	GC	367	74.4%	29.4%	NA	Not associated with age, sex, tumour location, stage or OS.	(25)
CLDN18.2	Japan	GC	262 (PT) 135 (LNM)	87.0% (PT) 80.0% (LNM)	51.5% (PT) 45.2% (LNM)	NA	Associated with Lauren type and grade of dedifferentiation	(26)
CLDN18	South Korea	GC	134	48.5%	NA	Down	Associated with perineural invasion.	(27)
CLDN18.2	Germany	GC and GEJC	481	42.2%	27.4%	Down	Associated with mucin phenotype, EBV status, the integrin αvβ5, the EpCAM extracellular domain EpEX, and lysozyme	(28)
CLDN18.2	Germany	GC and GEJC	381 (PT) 146(LNM) 36 (M)	53.0%(PT) NA(LNM) NA (M)	17.1%(PT) 26.7%(LNM) 16.7% (M)	NA	Not associated with age, sex, tumour location, stage or OS.	(29)
CLDN18	Italy	GC and GEJC	510 (PT) 132 (LNM)	61.6% (PT) 55.3% (LNM)	29.4% (PT) 34.1% (LNM)	NA	Associated with tumour localisation, Lauren type and EBV-positive status	(30)
CLDN18	Italy	GC and GEJC	350 (PT) 41 (M)	70.6% (PT) 65.9%(M)	33.4% (PT) 46.3% (M)	NA	Associated with age, EBV status, stage, peritoneal involvement and lower incidence liver metastasis; not associated with OS	(31)
CLDN18	Brazil	GC	349	17.5%	NA	NA	Associated with lower venous invasion and high-HER2; not associated with DFS, OS	(32)
CLDN18.2	Germany	Pancreatic neoplasms	202 (PT) 49 (LNM) 35 (LM) 24 (NT)	54.0% (PT) 69.4% (LNM) 65.7% (LM) 0 (NT)	50.0%(PT) 65.3% (LNM) 65.7% (LM) 0 (NT)	Up	Associated with LNM	(33)
CLDN18	Japan	PDAC	156	69.9%	50.0%	Up	Associated with differentiation stage	(34)
CLDN18	Japan and Finland	PDAC	111	70.3%	50.5%	Up	Associated with differentiation stage	(35)
CLDN18.2	Chinese	PDAC	93 (PT) 13 (NT)	94.6% (PT) 0.0% (NT)	56.8% (PT) 0.0% (NT)	Up	Associated with LNM, DM, nerve invasion, stage; not associated with OS	(36)
CLDN18.2	Chinese	PDAC	302	56.5%	NA	NA	Associated with sex, smoking, abdominal pain, jaundice, differentiation, common bile duct invasion, and M stage, improved survival; not an independent prognostic predictor	(37)
CLDN18	Japan	Intrahepatic bile duct carcinoma	83	43.4%	22.9%	Up	Associated with LNM and poor OS	(38)
CLDN18	Japan	Extrahepatic bile duct carcinoma	99	89.9%	46.5%	Up	Not associated with age, sex, tumour location, stage or OS.	(38)
CLDN18.2	Japan	Sporadic colorectal adenocarcinoma	56	5.4%	5.4%	NA	NA	(39)
CLDN18.2	Japan	Colitis-associated colorectal adenocarcinoma	56	26.8%	26.8%	NA	Associated with LNM; associated with MUC5AC	(39)
CLDN18	China	Advanced gastric signet-ring cell carcinoma	105	95.2%	95.2%	Up	Associated with age, not a prognostic risk factor for OS; related to GRIN2A mutation	(40)

OS, overall survival; DFS, disease-free survival; GC, gastric cancer; GEJC, gastroesophageal junction cancer; PDAC, pancreatic ductal adenocarcinoma; PT, primary tumour; LNM, lymph node metastasis; LM, liver metastasis; M, metastatic disease; NT, normal tissue; NA, not applicable.

Up to present, for CLDN18.2 detection modalities, the vast majority of clinical studies use tissue samples to detect its protein expression by immunohistochemistry, including all studies in **Table 1**. Besides, Sanada et al. also tested CLDN18.2 mRNA expression by reverse transcription (RT)-polymerase chain reaction (PCR) (41). In addition, one study innovatively designed a CLDN18.2 molecular beacon (MB) with high resolution and selectivity to distinguish CLDN18.2 RNA expression in circulating tumour cells (CTCs). They compared immunohistochemistry to MB data in 10 GC patients and verified that the concordance in the expression of CLDN18.2 between CTCs and tissue biopsy is 100% (negative: 3 vs 3; positive: 7 vs 7) (42). Thus, CTCs detection in peripheral blood, a non-invasive and method, may be a promising approach for evaluating CLDN18.2 expression and subsequently contribute to timely treatment for patients diagnosed with CLDN18.2 highly expressed GC. Further investigations are needed for the other courses of tumours like prognosis and the tests for more patients.

The CLDN18.2 protein is a highly selective gastric lineage marker expressed in short-lived differentiated cells but not the stem cell zone of stomach mucosa (43). In addition to its orthotopic expression, frequent ectopic activation of CLDN18.2 was detected in pancreatic, oesophageal, biliary tract, ovarian, and lung tumours, whereas downregulation was found in gastric cancer (GC) and sarcoma (gastric stromal tumour) tissues compared to their corresponding normal tissues (38, 43–45). It is worth noting that CLDN18.2 expression is decreased but still stable during gastric malignant transformation. Moreover, CLDN18.2 activation in metastatic lesions (lymph node and liver metastases) is comparable to that in primary lesions (26, 30, 31). These findings are of great significance, as they indicate that not only those with locally advanced disease but also those with metastatic cancer may be candidate patient populations for clinical trial targeting CLDN18.2.

CLDN18 expression is associated with histological subtype in GC. In GCs grouped by the Lauren subtype, CLDN18.2 expression was significantly higher in diffuse-type GCs (26, 30). In terms of the mucin phenotype, samples with the intestinal phenotype predominantly showed downregulation of CLDN18.2, suggesting that the loss of CLDN18 is involved in the pathogenesis of tumours of the intestinal phenotype and indicating that its expression can be used as a marker for the gastric phenotype (28, 41, 46).

In the relationship between CLDN18.2 expression and clinicopathological factors, Jun et al. showed a significant inverse correlation between claudin-18 expression and perineural invasion (27). In addition, a study of Japanese patients with gastric adenocarcinoma revealed that moderate-to-strong CLDN18.2 expression positively correlated with a higher grade of dedifferentiation (59.0% vs. 37.7% for G3 and G1/2 tumours, respectively; P = 0.005) (26). However, Soini et al. identified an inconsistent result with the previous study that positive CLDN18 expression is associated with a higher differentiation stage (35). In the pancreas, a correlation analysis revealed that lymph node-positive tumours had significantly higher CLDN18.2 expression indicating by the positive cell fractions and membrane staining intensities (33).

Claudin-18 has also been reported to be correlated with survival in GC patients. Jun et al. suggested the loss of claudin-18 as an independent indicator of a poor prognosis in patients with GC, as patients with claudin-18 expression had longer overall survival (OS) than those without claudin-18 expression (27). Sanada et al. also indicated that downregulation of claudin-18 was correlated with poor survival in patients with advanced GCs (41). However, in pancreatic cancer (PC), although there was an association between CLDN18.2 expression and lymph node metastasis (LNM) and dedifferentiation stage, as mentioned above, no correlation between CLDN18.2 expression and patient survival was found (33–36). In endocervical adenocarcinoma, gastric immunophenotypes (CLDN18.2) were identified as an independent predictor of poorer progression-free survival (PFS) (47).

Moreover, CLDN18-ARHGAP26/6 fusions have been identified in GCs (48). Tanaka et al. analysed 254 cases of GC (172 diffuse-type and 82 intestinal-type) using RT-PCR and situ hybridisation (FISH) and identified 26 fusion-positive cases (26/254, 10.24%), 22 of which were diffuse GCs (22/172, 12.79%), indicating that the CLDN18-ARHGAP26/6 fusion was specific to diffuse-type GCs (49). However, Hashimoto et al. found that intestinal-type adenocarcinoma with anastomosing glands, a genetically distinct group of intestinal-type adenocarcinoma, also frequently had CLDN18-ARHGAP fusion (50). Shu et al. found that the frequency of CLDN18-ARHGAP fusion is 18.25% in signet-ring cell carcinoma, and the CLDN18-ARHGAP26/6 fusion is associated with signet-ring cell content, age at diagnosis, female/male ratio, and TNM stage. The survival rate of patients with CLDN18-ARHGAP26/6 fusion is worse, and patients with this fusion obtain no benefit from oxaliplatin/fluoropyrimidine-based chemotherapy (51). Further studies have identified significant drug resistance acquired in CLDN18-ARHGAP26-introduced cell lines (51). Matsusaka et al. found that the combination of CDH17 and CLDN18 demonstrates homogeneous and robust expression in more than 90% of GC cases, and their coupling is helpful to detect and localise GC metastasis *in vivo*, which provides a possibility to overcome the challenge of intratumoural heterogeneity (52). Nakayama et al. found that the CLDN18‐ARHGAP fusion rate was 15.1% in 146 patients with GC under the age of 40. Among them, 18 cases (12.3%) were CLDN18-ARHGAP26 fusions, and 2 cases (1.4%) were CLDN18-ARHGAP6 fusions. Two new fusion types, CLDN18‐ARHGAP10 and CLDN18‐ARHGAP42, were identified. Moreover, patients with fusion were identified to more likely to have large tumours, LNM, and advanced stage and to have exhibited significantly lower OS by Kaplan‐Meier analysis. These observations indicate that the CLDN18‐ARHGAP fusion is enriched in younger age‐onset GCs, and its presence could contribute to their aggressive characteristics (53). Yao et al. demonstrated that the CLDN18-ARHGAP26 fusion mediates epithelial disintegration and contributes to GC by loss of CLDN18 and gain of ARHGAP26 functions (54). The CLDN18-ARHGAP fusion is one of the molecular characteristics of diffuse GC and is also an independent prognostic risk factor for GC. In addition, it is related to multiple clinicopathological characteristics, including age, sex, chemotherapy resistance, LNM and tumour stage. However, further exploration is required to elucidate the mechanism of the CLDN18-ARHGAP fusion gene and identify potential targeted therapeutic strategies.

## 4 Regulation of CLDN18 expression and its functions in tumours

It is universally acknowledged that a comprehensive and systematic understanding of target molecules’ functions in tumours is rather essential, partly because it can help to correctly apply therapeutic strategies to treat tumour patients. Studies for molecular regulatory mechanism include upstream mechanism study aiming to explore the pathways and molecules that regulate target molecules, and downstream mechanism study aiming to explore the pathways and molecules that are regulated by target molecules. The focus is on the latter in most cases. However, understanding the upstream mechanism is also very important. A comprehensive understanding of upstream regulatory factors can better clarify the source of target molecules and the causes of their changes, providing more insights for antitumour therapies that target molecular markers. In this section, we review the factors that regulate CLDN18 expression, as well as the roles of CLDN18 in cancers of the GI tract.

### 4.1 Regulation of CLDN18 expression

Previous studies have shown that carbonic anhydrase 9 (CAIX), phorbol 12-myristate 13-acetate (PMA), c-jun, epidermal growth factor (EGF) and RAS can upregulate the expression of CLDN18 (15, 55–57) and that interleukin-1β (IL-1β), hyperoxia, STE20/SPS1-related proline/alanine-rich kinase (SPAK) can inhibit CLDN18 expression (57, 58). The possible mechanisms are summarised as follows:

#### 4.1.1 Methylation of CpG islands

**Figure 2:**
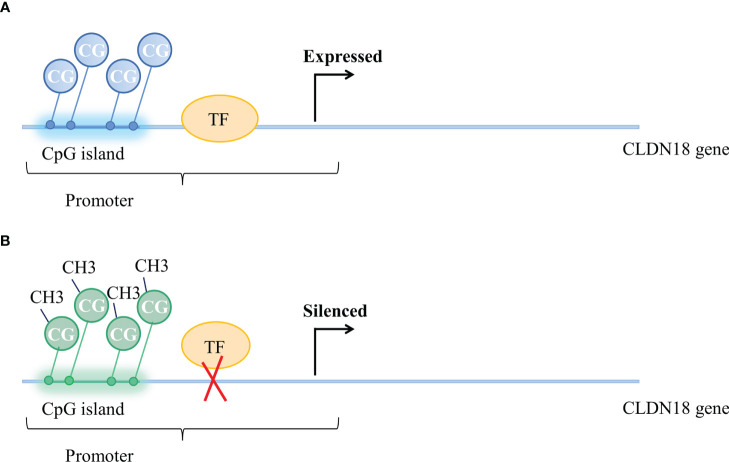
Methylation status of CpG islands and CLDN18 gene expression. **(A)** Unmethylated CpG island and expressed CLDN18.2 gene. **(B)** Methylation of CpG islands blocks the binding of transcription factors to the CLDN18.2 promoter, resulting in CLDN18.2 gene silencing.

Methylation of CpGs in promoter regions was suggested as a mechanism for the regulation of CLDN18 expression. The methylation status of CpG is correlated with CLDN18 gene expression (**Figure 2**). According to an analysis of data from the LUAD patient cohort of TCGA, CLDN18.1 mRNA expression is inversely correlated with the methylation of its promoter CpG island (20). It has been validated that *in vitro* methylation of the CLDN18.1 promoter can strongly inhibit the transcription of a linked luciferase reporter, suggesting that methylation of the CpG island may suppress the binding of transcription factors to the CLDN18.1 promoter and attenuate CLDN18.1 expression (20). The Claudin-18a1 promoter contains two binding sites of T/EBP/NKX2.1, which is a homeodomain-containing transcription factor (59). T/EBP/NKX2.1 binds to the claudin-18a1 promoter and promotes the transactivation of claudin-18a1 expression, and mutation of each motif attenuates claudin-18a1 promoter activity by approximately one-half, while mutation of both sites nearly abolishes the transcriptional activity of the gene (60). In addition, Sahin et al. indicated that methylation of CpG islands could completely prevent the transcription factor CREB from binding the promoter regions of CLDN18.2, which is required to activate CLDN18.2 transcription (43).

#### 4.1.2 PKC pathway and ERK/MAPK pathway

Protein kinase C (PKC) is an effector in the G protein-coupled receptor system and can promote the phosphorylation of a variety of proteins involved in many physiological, biochemical and pathological processes of cells, including the induction of cell activation and proliferation, differentiation, motility, and survival. In addition, PKC affects transcription factors in the nucleus and thus plays an important role in the regulation of gene expression (61).

**Figure 3:**
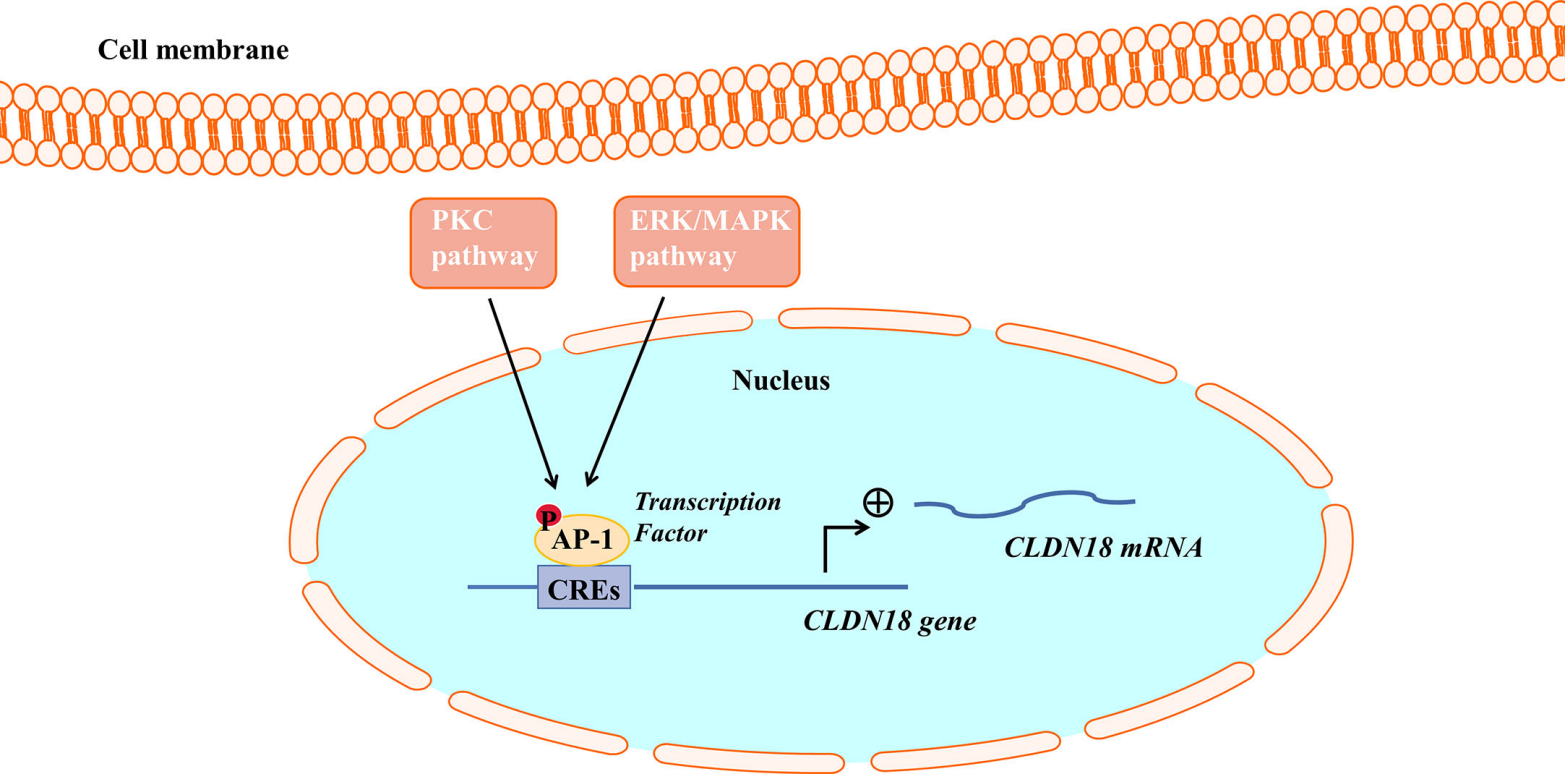
The PKC pathway and ERK/MAPK pathway regulate CLDN18.2 expression. AP-1 could bind to cis-regulatory elements (CREs) of the CLDN18.2 promoter, increasing the transcriptional activity of CLDN18.2. The PKC and ERK-MAPK pathways enhance the transcription of CLDN18.2 mRNA by stimulating the phosphorylation and activation of AP-1.

Studies have found that the PKC pathway and extracellular signal-related kinase (ERK)/Mitogen-activated protein kinase (MAPK) pathway are involved in regulating the expression of CLDN18.2. The PKC pathway and ERK/MAPK pathway regulate the expression of CLDN18.2 by regulating an intracellular transcriptional activator (**Figure 3**). PMA is the most commonly used phorbol ester; it binds to and activates PKC, causing a wide range of effects in cells and tissues. Studies have demonstrated that when treated with PMA, the mRNA and protein expression of CLDN18.2 in pancreatic and GC cell lines is upregulated. However, a PKC inhibitor completely repressed the upregulation of CLDN18.2 induced by PMA, indicating that the PKC pathway is involved in regulating the expression of CLDN18.2 in carcinogenesis (34, 55, 62). The SPAK-p38 MAPK signalling pathway is also involved in hyperoxia-induced barrier dysfunction of the alveolar epithelium by suppressing the expression of claudin-18 (57).

Activator protein (AP)-1 is an intracellular transcriptional activator and is required for cell-specific gene expression. AP-1 sites were shown to bind to cis-regulatory elements of the CLDN18a2 promoter, increasing the transcriptional activity of CLDN18.2 (55). It was further found that PMA induced the PKC and ERK-MAPK pathways and stimulated the phosphorylation and activation of AP-1, thereby enhancing the transcription of CLDN18.2 mRNA (55).

#### 4.1.3 HER2/HER3 signalling pathway

**Figure 4:**
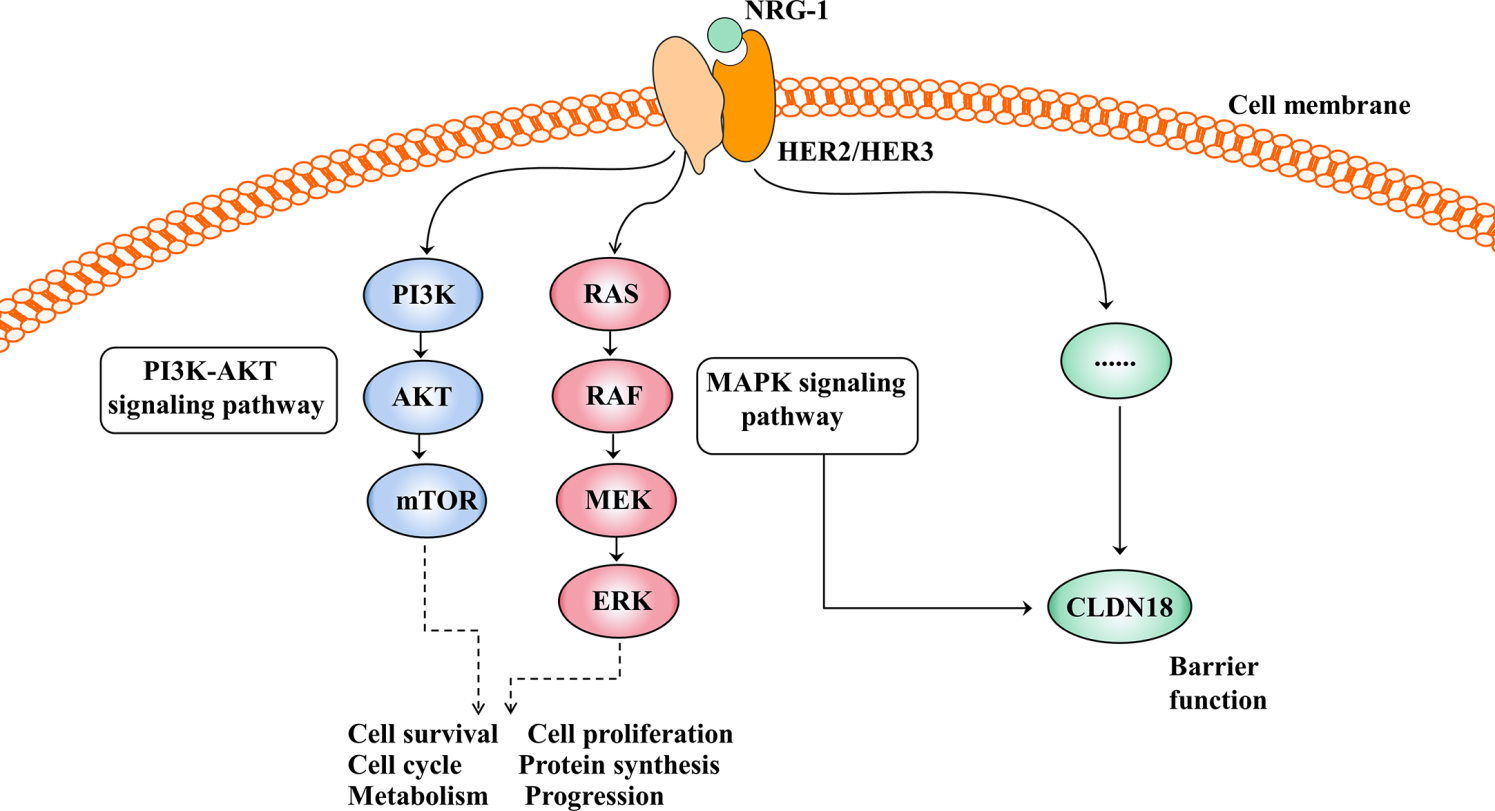
Activation of the HER2/HER3 signalling pathway and its effect after activation. The PI3K-AKT and MAPK signalling pathways are the two main signal transduction pathways downstream of HER2/3 heterodimerisation activation and are involved in cell growth, cell proliferation, the cell cycle, protein synthesis and so on. In addition, the HER2/HER3 signalling pathway regulates barrier function by regulating the expression of CLDN18, but the mechanism is unclear.

The HER family consists of four membrane-bound tyrosine kinase receptors with similar structures: HER1 (EGFR), HER2, HER3, and HER4 (63). HER2 has no specific ligand, so only when it partners with another HER family member can it be activated. HER3 is the receptor for the ligand neuregulin-1 (NRG-1), but HER3 lacks intrinsic signalling properties (64). Upon NRG-1 binding, HER3 heterodimerises with HER2, resulting in activation of the downstream signalling pathway and involvement in normal cell growth and development as well as in the pathogenesis of epithelial malignancies (**Figure 4**) (65).

The HER2/HER3 signalling pathway is also involved in barrier function by regulating the expression of claudin18 (**Figure 4**). Finigan et al. revealed that the NRG-1-HER2/3 signalling pathway participates in IL-1β-mediated lung barrier dysfunction (66). In another study to identify potential corresponding pathways by which IL-1β promotes the development of acute respiratory distress syndrome (ARDS), Ma et al. showed that the HER2 blocker lapatinib blocked the effect of IL-1β on the downregulated expression and cell membrane localisation of claudin18, suggesting that IL-1β suppresses the protein expression and cell membrane localisation of claudin18 by activating the HER2/HER3-associated signalling pathway (58).

#### 4.1.4 MicroRNA

**Figure 5:**
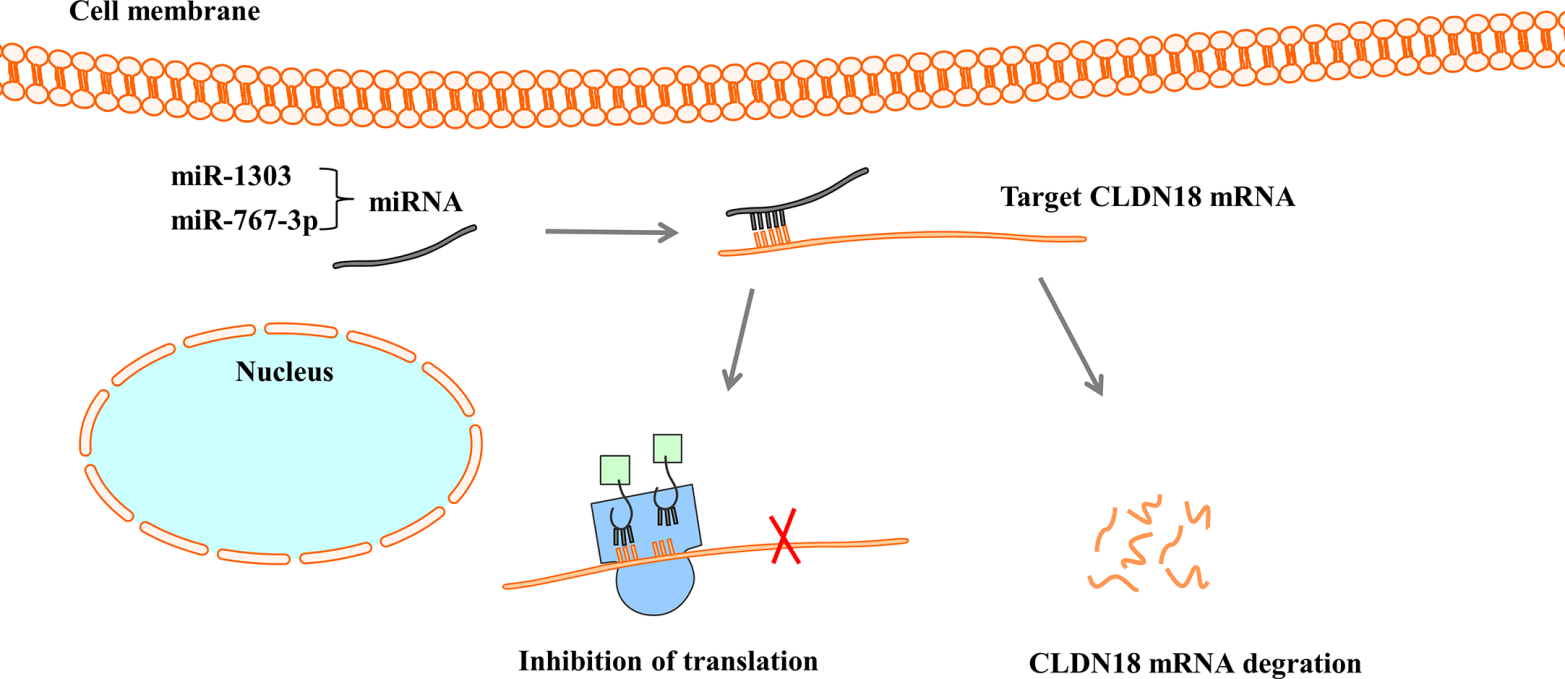
MiRNAs regulate the expression of CLDN18. MiRNAs bind with 3’-UTR of CLDN18 mRNA, resulting in the degradation of mRNA or the inhibition of translation.

MicroRNAs (miRNAs) are a large family of small endogenous regulatory RNAs that regulate posttranscriptional regulators of gene expression (67, 68). MiRNAs target both messenger RNA (mRNA) degradation and suppression of protein translation based on sequence complementarity between the miRNA and its targeted mRNA (69). MiRNAs regulate the expression of CLDN18 by binding to CLDN18 mRNA (**Figure 5**). Western blot analysis and luciferase reporter assays showed that miR-1303 could markedly attenuate the expression of claudin-18 by binding to the putative binding sites in the CLDN18 mRNA 3’-UTR (70). Wan et al. reported that miR-767-3p targets CLDN18, thus inhibiting tumour cell proliferation, migration, and invasion, providing a promising therapeutic target for LUAD (71).

The findings to date indicate that CLDN18 is regulated at the transcriptional level through the methylation of CpG islands, the PKC pathway, the ERK/MAPK pathway, and the HER signalling pathway and is regulated at the posttranscriptional level by miRNAs, providing new approaches for CLDN18-based cancer therapy.

### 4.2 Dichotomous roles of CLDN18 in cancers

CLDN18 has dichotomous roles in cancers: it can act as a tumour promoter or suppressor depending on the tumour type. CLDN18 plays a tumour suppressor role in gastric and lung cancers but functions as a tumour promoter in other cancers of the GI tract, including oesophageal, pancreatic, colorectal and bile duct cancers, which is consistent with the ectopic expression pattern described above. Although many studies have explored the different roles of CLDN18 in tumours, only a few articles have clarified its mechanisms.

#### 4.2.1 The tumour suppressor role of CLDN18

Claudin-18 expression is significantly decreased in GC tissue compared to surrounding gastric normal mucosa or intestinal metaplasia. In early GC lesions removed *via* endoscopic mucosal resection or endoscopic submucosal resection, Oshima et al. evaluated the expression patterns of claudin-18 and Ki-67, a marker of cell proliferation, and then identified an inverse correlation between them at the invasive front. Subsequent experiments in GC cell lines further confirmed that knockdown of Claudin-18 could significantly promote cell proliferation and invasion, indicating a tumour suppressor role for CLDN18 (14). Hagen et al. showed that H. pylori infection in mice attenuates the expression of CLDN18 early in GC development, which is similar to the results in human patients. Mechanistically, the loss of CLDN18 promotes the rapid development of cancer cells in the stomach of mice, possibly because CLDN18 is a top regulator and effector of the network that regulates the cytokine, stemness, Wnt and Notch signalling pathways (16). In the lung, Zhou et al. identified that CLDN18 could reversely regulate the nuclear localisation and activity of the downstream transcriptional coactivator Yes-associated protein (YAP) and thus promotes the proliferation of AT2 cells in normal lung as well as that of tumour cells found in lung adenocarcinomas thought to originate from AT2 cells (17, 72). Given that expression of the lung-specific isoform (CLDN18.1) is markedly decreased in LUAD and that aged Cldn18 -/- mice have an increased propensity to develop LUAD, CLDN18.1 was assumed to have a tumour suppressor role in LUAD, and this was validated by Luo et al. They demonstrated that CLDN18.1 expression is inversely correlated with promoter methylation and with LUAD patient mortality. In addition, the restoration of CLDN18.1 expression in LUAD cells that have lost expression markedly attenuated malignant properties, including xenograft tumour growth *in vivo* as well as cell proliferation, migration, invasion and anchorage-independent colony formation *in vitro*. Further mechanistic studies have shown that CLDN18.1 attenuates malignancy by inhibiting insulin-like growth factor-1 receptor (IGF-1R)/AKT and the YAP/TAZ/AKT axes (20). Studies also found that claudin-18.1 could suppress the proliferation and motility of lung epithelial cells by inhibiting the phosphatidylinositol-3 kinase (PI3K)/pyruvate dehydrogenase kinase-1 (PDK1)/Akt signalling pathway (19, 73). In addition, downregulating the CLDN18-dependent ZO-2 expression in CLDN18/A549 cells enhanced the expression and activity of matrix metalloproteinase 2 (MMP2), resulting in the promotion of cell migration (18).

#### 4.2.2 The tumour promoter role of CLDN18

The cancer-promoting effects of CLDN18 are mostly manifested in cancers with ectopic activation of this factor. Three distinct epithelial lesions—pancreatic intraepithelial neoplasias (PanINs), intraductal papillary mucinous neoplasms (IPMNs), and mucinous cystic neoplasms (MCNs)—are recognised as precursors of PDAC (34). CLDN18 is a marker for the early carcinogenetic process in PDAC because all three types of precursor lesions (PanIN, IPMN, and MCN) exhibit frequent immunoreactivity for CLDN18. In the oesophagus, hybrid stomach-intestinal chromatin states, including those involving CLDN18, underlie human Barrett’s metaplasia (74). The change from a CLDN18-deficient tight junction in healthy squamous epithelium from subjects without oesophageal disease to a Cldn-18-rich tight junction in specialised columnar epithelium of Barrett’s oesophagus contributes to preventing the squamous epithelium from being attacked and destroyed by luminal acid, thereby promoting tumourigenesis (15, 75). For bile duct carcinoma, it was found that the knockdown of claudin-18 expression or an antibody specific to claudin-18 can significantly attenuate the proliferation, invasion and *in vivo* tumourigenesis of bile duct adenocarcinoma cells. Researchers further revealed that the EGFR pathway and active RAS can induce claudin-18 expression by activating ERK1/2. Enhanced claudin-18 expression subsequently activates ERK1/2. Thus, they form a positive feedback loop, playing a role in the promotion of malignancy in the bile duct (15). These above research findings preliminarily suggested its tumour promoter role in cancers with ectopic activation of CLDN18. Nevertheless, more comprehensive, in-depth studies are required as the mechanism evidence exploration yet is shallow.

## 5 Research progress on drugs targeting CLDN18.2

Researchers have successively developed more specific or effective antibodies to detect CLDN18.2 expression (76, 77) or target it as a therapeutic candidate for tumours (78–82). In the field of therapies targeting Claudin18.2, there are many drugs in the clinical development stage, including monoclonal antibodies, CAR-T cells, bispecific antibodies and ADCs. Among them, several clinical studies of monoclonal antibody therapy and CAR-T therapy have disclosed clinical trial data to date. The latter two emerging drug types, ADCs and bispecific antibodies, have been approved for clinical testing in recent years, and studies are still ongoing. In contrast to previous clinical trials that only assessed the safety and efficacy of CLDN18.2-targeted therapies in populations with advanced gastric and oesophagogastric junction cancers, recently, approved clinical trials have also expanded to include patients with other advanced solid tumours, mostly tumours of the digestive system, including PC, colorectal cancer, intrahepatic cholangiocarcinoma, and extrahepatic bile duct cancer. The CLDN18.2 clinical trials with disclosed results are shown in **Table 2**; ongoing clinical trials of CLDN18.2 are shown in **Table 3**.

**Table 2:** CLDN18.2 clinical trial with disclosed results.

NCT ID	Country	Enrollment	Interventions	Category	Tumour	Cut-off value of CLDN18.2	Outcomes	Phase	References
NCT00909025	German	15	IMAB362	mAb	Advanced GAC, GEJC	Any SI and any TCs	–	I	(83)
NCT01197885	Bulgaria	54	IMAB362	mAb	Advanced GAC, GEJC and EAC	≥2+ SI and ≥50% TCs	OS; mPFS; ORR 9%	IIa	(84)
NCT01671774	German	32	IMAB362 + ZA + IL-2	mAb	Advanced GAC, GEJC and EAC	NA	mPFS 12.7 weeks; mOS 40 weeks	I	(85)
NCT01630083	Bulgaria	246	IMAB362 + EOX	mAb	Advanced GAC, GEJC and EAC	≥2+ SI and ≥ 40% TCs	mPFS 7.5 months; mOS 13.0 months; ORR 29%	II	(86)
NCT03505320	United States	21	IMAB362 + mFOLFOX6	mAb	Advanced GAC, GEJC	Any SI and ≥75%TCs	mPFS 13.7 months; ORR 63.2%	II	(87)
NCT03159819	China	12	CAR-CLDN18.2 T Cells	CAR-T	Advanced GAC, PC	NA	mPFS 130 day; ORR 33.3%	I	(88)
NCT03874897	China	37	CT041	CAR-T	Cancer of digestive system	≥2+ SI and ≥40% TCs	mOS 7.6 months; ORR 48.6% DCR 73.0% (GC patients: ORR 57.1% DCR 75.0%)	I	(89)

mAb, monoclonal antibody; bsAb, bispecific antibody; ADC, antibody–drug conjugate; CAR-T, chimeric antigen receptor T; IMAB362, zolbetuximab; SI, staining intensity; TCs, tumour cells; GC, gastric cancer; GAC, gastric adenocarcinoma; GEJC, gastroesophageal junction cancer; EAC, oesophageal adenocarcinoma; PC, pancreatic cancer; OS, overall survival; PFS, progression-free survival; mOS, median overall survival; mPFS, median progression-free survival; ORR, objective response rate; DCR, disease control rate.

**Table 3:** Ongoing clinical trial of CLDN18.2.

NCT ID	Country	Enrollment	Interventions	Category	Tumour	Cut-off value of CLDN18.2	Outcomes	Phase	Status
NCT03504397	United States	550	IMAB362 + mFOLFOX6 vs. Placebo + mFOLFOX6	mAb	Advanced GC, GEJC	≥ 75%TCs	P: PFS S: OS, ORR, DOR, AEs, HRQoL, PK, Immunogenicity	III	Recruiting
NCT03653507	United States	500	IMAB362 + CAPOX vs. Placebo + CAPOX	mAb	GAC, GEJC	NA	P: PFS S: OS, ORR, DOR, AEs, PK	III	Active, not recruiting
NCT03816163	United States	369	IMAB362 + Nab-P + GEM vs. Nab-P + GEM	mAb	Advanced PC	≥ 75%TCs	P: DLT, OS, AEs, SAEs S: PFS, ORR, DCR, DOR, PK, HRQoL	II	Recruiting
NCT03505320	United States	116	IMAB362 vs. IMAB362 + mFOLFOX6 vs. IMAB362 + Pembrolizumab vs. IMAB362 + mFOLFOX6 + nivolumab	mAb	Advanced GAC, GEJC	NA	P: ORR S: PK, AEs, PFS, Immunogenicity, DCR, DOR, ORR, OS	II	Recruiting
NCT04086758	China	12	IMAB362	mAb	GAC, GEJC	NA	P: PK, AEs S: ORR, DCR, PFS, DOR, OS	I	Completed
NCT04495296	China	210	TST001	mAb	Advanced solid tumours	≥ 2+SI and ≥ 40%TCs	P:DLT, MDT, SAEs, S:PK, ORR, DOR, CBR, PFS, Immunogenicity	I	Recruiting
NCT04396821	United States	114	TST001	mAb	Advanced solid tumours	NA	P: AEs, SAEs, MTD, DLTs S: ORR, DOR, CBR, PFS, PK, Immunogenicity	I	Recruiting
NCT05190575	China	40	TST001	mAb	Advanced biliary tract cancer	NA	P: ORR S: PFS, OS, DOR, DCR, AEs	II	Not yet recruiting
NCT05065710	United States	162	ZL-1211	mAb	Advanced solid tumours	NA	P:MTD or MAD, AEs, CBR S:PK, DOR, Immunogenicity	I/II	Recruiting
NCT04683939	United States	96	BNT141	mAb	Advanced solid tumours	≥ 2+SI and ≥ 50%TCs	P:EAEs, DLT, MDT S:PK, ORR, DCR, DOR	I/IIa	Recruiting
NCT05008445	China	265	LM-102 vs. LM-102+SOC	mAb	Advanced solid tumours	NA	P: DLT, AEs, RP2D, MTD S: Immunogenicity, PK, DCR, DOR, PFS	I/II	Recruiting
NCT04671875	China	228	MIL93	mAb	Advanced solid tumours	NA	P: AEs, SAEs S: PK, ORR, DOR, PFS, Immunogenicity	I	Recruiting
NCT04400383	China	197	AB011	mAb	Advanced solid tumours	NA	P: AEs, SAEs, DLT S: PK, ORR, DOR, PFS, OS, Immunogenicity	I	Recruiting
NCT04404595	United States	110	CT041	CAR-T	Advanced GAC, GEJC, PC	NA	P: AEs, SAEs, MDT, DLT S: DOR, DCR, OS, PFS	Ib	Recruiting
NCT04581473	China	102	CT041	CAR-T	GAC, GEJC and PC	NA	P: AEs, SAEs, MTD, DLTs, ORR S: DCR, DOR, PFS, OS	Ib/II	Recruiting
NCT03874897	China	123	CAR-CLDN18.2 T cells	CAR-T	Advanced GAC, GEJC	≥2+ SI and ≥40% TCs	P: DLT, MTD S: PK, AEs, PFS, OS, DOR, ORR, DCR	I	Recruiting
NCT04467853	China	34	LCAR-C18S	CAR-T	Advanced GAC, GEJC	NA	P:DLT, TEAEs, RP2D, CAR-T positive cell concentration S:ORR, DOR, OS, PFS, DCR, TTR	I	Recruiting
NCT04966143	China	30	LY011	CAR-T	Advanced PC	NA	P:ORR S:PFS, DCR, DOR, OS, SAEs	I	Recruiting
NCT05161390	China	128	LM-302	ADC	Advanced solid tumours	NA	P: DLT, AEs, RP2D, MTD S:PK	I/II	Enrolling by invitation
NCT05001516	United States	42	LM-302	ADC	Advanced solid tumours	≥1+SI and ≥ 10%TCs	P: DLT, AEs, MTD, Vital Signs, ECG, Abnormal Clinical Laboratory Test Results S: Immunogenicity, PK, PFS, ORR, DCR	I/II	Recruiting
NCT05009966	China	272	SYSA1801	ADC	Advanced solid tumours	≥ 2+SI and ≥ 40%TCs	P:DLT, MDT, AEs, SAEs S:PK, ORR, DCR, PFS, Immunogenicity	I	Recruiting
NCT04914117	Australia	33	RC118	ADC	Advanced solid tumours	NA	P:DLT, MDT, AEs S:PK, ORR, PFS, DCR, Immunogenicity	I	Active, not recruiting
NCT05043987	China	72	CPO102	ADC	PC, GC, GEJC	≥ 2+SI and ≥ 50%TCs	P:MDT, DLT S: PK, TEAEs, ORR, Immunogenicity	I	Not yet recruiting
NCT04805307	China	162	CMG901	ADC	GC, GEJC, PC	NA	P:DLT, TEAEs, SAEs, ORR, RP2D S: PK, DCR, DOR, PFS, OS, ORR	I	Recruiting
NCT04856150	China	66	Q-1802	bsAb	Advanced solid tumours	NA	P: DLTs S: PK, TEAEs, ORR, DOR, DCR, OS, PFS,	I	Not yet recruiting
NCT04260191	United States	16	AMG 910	bsAb	GAC, GEJC	NA	P: DLT, MTD S: ORR, PK, PFS, DOR, DCR, OS	I	Completed

mAb, monoclonal antibody; bsAb, bispecific antibody; ADC, antibody–drug conjugate; CAR-T, chimeric antigen receptor T; IMAB362, zolbetuximab; SI, staining intensity; TCs, tumour cells; GC, gastric cancer; GAC, gastric adenocarcinoma; GEJC, gastroesophageal junction cancer; GEJC, gastric oesophageal junction cancers; P, primary outcomes; S, secondary outcomes; PC, pancreatic cancer; OS, overall survival; PFS, progression-free survival; ORR, objective response rate; DLTs, dose-limiting toxicities; AEs, adverse events; SAEs, serious adverse events; TEAEs, treatment-emergent adverse events; MTD, maximum tolerated dose; RP2D, recommended phase 2 dose; PK, pharmacokinetic; DCR, disease control rate; DOR, duration of response.

### 5.1 Monoclonal antibody therapies

**Figure 6:**
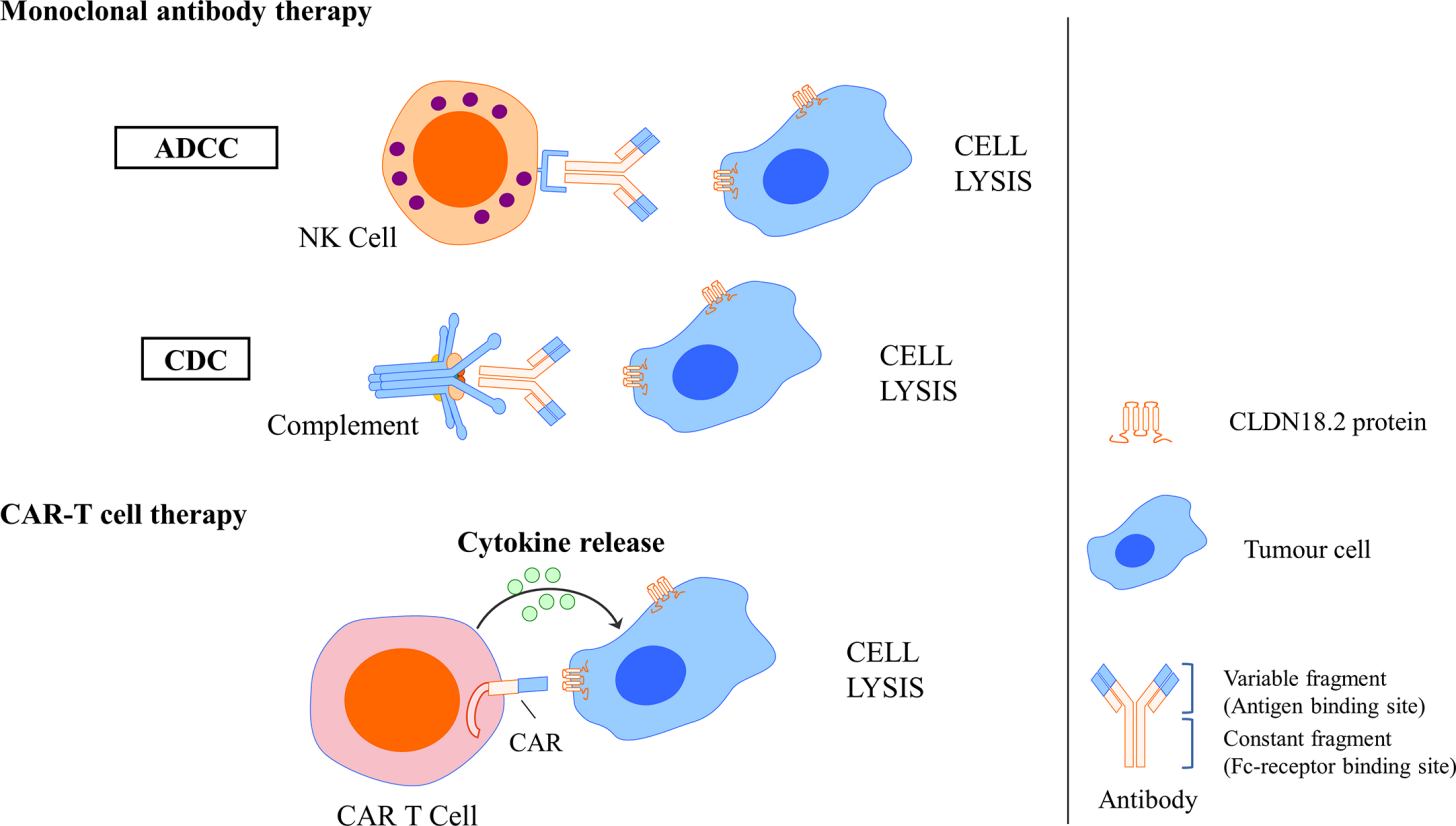
Mechanisms of CLDN18.2-targeted monoclonal antibody intervention and CAR T cell interaction. Monoclonal antibody could specifically bind to CLDN18.2 expressed tumour cells and induce ADCC and CDC, resulting in the cell lysis. CLDN18.2 CAR T cell could also specifically recognise CLDN18.2 expressed tumour cells and induce cytokine release, resulting in the cell lysis.

In a basic study investigating the antitumour activity and mechanism of IMAB362, Tureci identified that zolbetuximab bound with high specificity and affinity to human PC cells expressing CLDN18.2 and induced antibody-dependent cellular cytotoxicity (ADCC) and complement-dependent cytotoxicity (CDC), the amplitude of which directly correlated with CLDN18.2 levels, resulting in the lysis of human PC cells. *In vivo* experiments also showed that zolbetuximab slowed tumour growth, improved survival, and attenuated metastasis development (90). Mechanisms of CLDN18.2-targeted monoclonal antibody intervention are showed in **Figure 6**.

For clinical trials, to date, 5 studies that measured the efficacy and safety of the monoclonal antibody IMAB362 have disclosed data. Among them, two are phase I studies, which suggested that IMAB362 has good tolerability and safety (83, 85), and three are phase II studies indicating that IMAB362 alone and IMAB362 combined with chemotherapy are safe strategies and have antitumour activity in patients with CLDN18.2-positive advanced gastric adenocarcinoma (GA), gastroesophageal junction cancer (GEJC), and oesophageal adenocarcinoma (EAC) (84, 86, 87). In addition, compared with chemotherapy alone, the combination therapy group maintained good quality of life and low symptom burden for a longer period (91). However, there is a great difference in survival benefits among these 3 phase II studies. The overall response rate (ORR) and clinical benefit rate (CBR) of the clinical trials (NCT01197885) of IMAB362 alone were 9% and 23%, respectively (84); in the study (NCT01630083) of treatment with IMAB362 plus epirubicin, oxaliplatin and capecitabine (EOX) chemotherapy, the ORR was 29%, and the median progression-free survival (mPFS) and median overall survival (mOS) were 7.5 and 13.0 months, respectively (86); the study (NCT03505320) investigating IMAB362 + mFOLFOX6 chemotherapy showed that the ORR was 63.2% and the mPFS was 13.7 months (87). The combination of the monoclonal antibody and chemotherapy had better survival benefits than monoclonal antibody alone. The differences among the 3 studies may be affected by other factors, including the combined chemotherapy regimen, drug dose, population inclusion criteria and so on.

Apart from the antitumour activity in GC and GEJC as mentioned above, IMAB362 is also suggested as an antibody for pancreatic cancers expressing CLDN18.2. A phase 2, open-label, randomised study (NCT03816163) of a large group of 369 patients with claudin 18.2-positive, metastatic pancreatic adenocarcinoma, began in 2019 and is still ongoing (92). The purpose of this study is to assess the efficacy and safety of IMAB362 in combination with nab-paclitaxel and gemcitabine (Nab-P + GEM) as first-line treatment. The primary outcomes are dose limiting toxicity (DLT), OS and adverse events (AEs). The results of this large-scale clinical trial are worthy of public expectation. In any case, it will be instructive for selection of patients applying CLDN18.2-targed therapies.

### 5.2 CAR-T cell therapy

Cancer immunotherapy with CAR-T cells is a breakthrough therapy of the twenty-first century for the management of different malignancies, including lymphomas and leukaemias (93– 95). Despite the progress made in treating haematological malignancies, challenges still remain for the use of CAR-T-cell therapy to treat solid tumours due to immune effector cell-related neurotoxicity syndrome (ICANS), treatment-related death, and severe cytokine-release syndrome (CRS). Encouragingly, the efficacy and safety of CAR-T-cell treatments for solid tumours have been improved mainly through optimisation of T-cell characteristics and reversal of the immunosuppressive microenvironment (96, 97).

A basic study successfully developed humanised CLDN18.2-specific single-chain variable fragments (scFvs) specifically recognising CLDN18.2 but not CLDN18.1. Then, CLDN18.2-specific CAR T cells that comprise the CD28 costimulatory domain were generated using scFv as targeting components. In GC, an *in vitro* assay showed that these CLDN18.2-specific CAR T cells could efficiently produce cytokine and lyse CLDN18.2-positive cells but not CLDN18.2-negative cells, indicating that they had intrinsic target-dependent cytotoxic activity. Potent suppression of tumour growth was also observed in *in vivo* tumour models after CAR T-cell treatment, whether in a cancer cell line xenograft mouse model or in patient-derived xenograft (PDX) models. Researchers have also demonstrated that these CAR T cells persist well *in vivo* and infiltrate efficiently into tumour tissues. In addition, in an assessment of safety, CLDN18.2 CAR T cells did not induce apparent damage to normal gastric tissues or other organs. These results demonstrated that CLDN18.2-specific CAR T cells could be a promising treatment strategy for GC (78). Mechanisms of CLDN18.2-targeted CAR-T cell interaction are showed in **Figure 6**.

Two clinical phase I trials of CAR-T therapy have disclosed results. One study (NCT03159819) evaluated CAR-CLDN18.2 T cells in 12 subjects with CLDN18.2-positive metastatic gastric or pancreatic adenocarcinoma. The total ORR was 33.3%, and the median PFS was 130 days (88). Another study (NCT03874897) enrolled 37 patients with cancer of the digestive system, including 28 with GC, 5 with PDAC and 4 with other cancer types. The ORRs for all patients and patients with GC were 48.6% and 57.1%, respectively. The disease control rates (DCRs) for all patients and patients with GC were 73.0% and 75.0%, respectively (89). In addition, the overall ORR, median PFS and OS values for 18 patients with GC who failed at least 2 prior lines of therapy were 61.1%, 5.4 months and 9.5 months, respectively, indicating that CT041 also displayed a significantly improved efficacy compared to historical data (98). In general, CAR-CLDN18.2 T-cell therapy showed an acceptable safety profile and promising antitumour activities in patients with advanced CLDN18.2+ cancers of the digestive system, taking the lead in the global solid tumour CAR-T field.

In contrast to the previous studies that mostly enrolled postoperative tumour patients, clinical studies covering patients who are not ideal candidates for surgery are emerging, mainly for advanced solid tumours. This is conducive to the settlement of some weaknesses regarding the lack of data on the damage to target organs and other adverse reactions caused by CAR-T cells. We are looking forward to the exploration of the efficacy and adverse reactions, especially the occurrence of cytokine storms and their prevention and control measures in the context of CAR-CLDN18.2 T therapy after multiline treatment.

### 5.3 ADCs and bispecific antibodies

Monoclonal antibody-based targeted therapy against specific molecules has greatly improved treatment approaches for patients. However, the long-term efficacy of therapeutic antibodies is limited by resistance mechanisms (99). Bispecific antibodies (bsAbs) and ADCs are the most prominent products of engineering and modification approaches being devised and applied to the conventional immunoglobulin molecular format and can overcome the limitations of such antibodies and enhance their efficacy (100). They are being developed as innovative targeted antibody therapies that eliminate cancer by redirecting immune cells or delivering a potent cytotoxic payload to tumours (101, 102).

Zhu et al. produced bispecific compounds by combining CLDN18.2- and CD3-targeting arms through hinge mutations and produced ADCs by conjugating anti-CLDN18.2 antibody to the cleavable auristatin. They showed the anti-CLDN18.2 ADC and anti-CLDN18.2-CD3 bsAb can effectively inhibit the growth of GC (KATO-III/hCLDN18.2) and PC (BxPC3/hCLDN18.2) cells *in vitro* and the PDX model with high expression of CLDN18.2. In conclusion, this study offers preclinical proof-of-concept that targeting CLDN18.2 with an ADC or bispecific modality could be an effective therapy for the treatment of gastric and pancreatic cancer (79). One study conducted by Liang et al. designed anti-CLDN18.2-CD28 bsAbs by connecting two scFvs against CLDN18.2 and CD28. *In vivo* mouse tumour models, compared with anti-CLDN18.2, anti-CLDN18.2-anti-CD28 showed a greater increase in the percentage of CD8+ T cells and higher expression of CD69 on CD8+ T cells in tumour tissues as well as a greater reduction in tumour burden. Treatment with anti-CLDN18.2-anti-CD28 also produced more interferon-γ (IFN-γ) and tumour necrosis factor-α (TNF-α) in splenocytes and reduced the levels of immunosuppressive cells, including tumour-associated macrophages and myeloid-derived suppressor cells, in tumour tissues. Thus, it is suggested that anti-CLDN18.2-anti-CD28 could play a tumour-killing role by improving antitumour immune response and attenuating the suppressive tumour microenvironment (80). Obvious side effects or signs of toxicity after anti-CLDN18.2 ADC and anti-CLDN18.2-CD3/CD28 bispecific treatment, even in CLDN18.2-expressing gastric tissues, were not observed in the above two studies. These findings collectively suggest that therapies targeting CLDN18.2 with ADCs and bispecific antibodies were both effective and safe in mouse tumour models.

Clinical trials of ADCs and bispecific antibodies are still ongoing and have not disclosed results. AMG-910 is a CD3/CLDN18.2 bsAb and the first CLDN18.2 bsAb that entered clinical trials. A phase I study of AMG-910 (NCT04260191) is ongoing, with the aim of evaluating its safety and tolerability in patients with claudin 18.2-positive GA and GEJA and determining the maximum tolerated dose (MTD) and/or recommended phase 2 dose (RP2D). Q-1802 is a bsAb targeting CLDN18.2 and the immune checkpoint PD-L1. The ongoing phase I study of Q-1802 (NCT04856150) consists of two compartments: the dose-exploration stage and the dose-extension stage. The aim is to establish safety and tolerance of Q-1802 in patients with advanced solid tumours and to assess the pharmacokinetic characteristics and efficacy. Subsequently, several studies aiming to evaluate the safety and efficacy of other ADCs or bsAbs, including CMG-901 SYSA1801, LM-302 and RC118, for the treatment of GC, GEJC and PC, are also still in phase I.

## 6 Conclusions and perspectives

CLDN18.2 is restricted to normal gastric tissue and is downregulated in GC tissues but is highly activated in other tumour tissues, including pancreatic, oesophageal, biliary tract, ovarian, and lung tumours. In addition, CLDN18.2 still has high expression during gastric tumour formation and retained expression in metastatic lesions. Therefore, CLDN18.2 is suggested as an ideal target for tumour treatment, especially in cancers of the GI tract. Moreover, CLDN18.2 can act as an oncogene or a tumour suppressor gene in different tumour types, and its expression is modulated by the methylation status of CpG islands, PKC, ERK/MAPK, HER2/HER3 signalling pathways and microRNA.

From the disclosed data, monoclonal antibody and CAR-T therapies have shown good safety in CLDN18.2-positive gastric and pancreatic cancer populations and have achieved encouraging preliminary results in terms of clinical efficacy. In addition, we are very much looking forward to encouraging the results of ongoing clinical trials of long-researched or emerging drugs targeting CLDN18.2 in various tumours, which may provide several new potential methods and benefits for millions of people with CLDN18.2-positive tumours, including GC, PC, hepatocellular carcinoma, and bile duct cancer. Further attention should be given to the definition of populations with high CLDN18.2 expression that may benefit from treatment, the prediction of marker efficacy, the prevention and control measures for adverse reactions, the selection of treatment regimens for multitarget coexpression populations, and the combination of immunotherapy, which are all directions for future exploration. Subsequent clinical trials of CAR-T cells, ADCs, and bsAb drugs will undoubtedly provide more options for patients with high CLDN18.2 expression and poor efficacy with targeted drugs or advanced multiline treatment.

## Author contributions

XC and LY conceptualised the manuscript. JC, ZX, CH, SZ, and MZ collected the literature, JC and ZX collected the literature, wrote the manuscript and made the figures. XC and LY edited and made significant revisions to the manuscript. All authors contributed to the article and approved the submitted version.
